# Qualitative Analysis of the Heat Transfer in a Package of Square Steel Sections

**DOI:** 10.3390/ma17225412

**Published:** 2024-11-06

**Authors:** Rafał Wyczółkowski, Vazgen Bagdasaryan, Suren G. Aghbalyan, Gayane A. Vasilyan, Marek Gała

**Affiliations:** 1 Department of Production Management, Czestochowa University of Technology, Armii Krajowej 19, 42-200 Czestochowa, Poland; rafal.wyczolkowski@pcz.pl; 2 Institute of Civil Engineering, Warsaw University of Life Sciences—SGGW, Nowoursynowska 166, 02-787 Warsaw, Poland; 3 Faculty of Mining and Metallurgy, National Polytechnic University of Armenia, Teryan 105, Yerevan 0009, Armenia; metalsur@polytechnic.am (S.G.A.); gayavasi@gmail.com (G.A.V.); 4 Institute of Electric Power Engineering, Czestochowa University of Technology, Armii Krajowej 17, 42-200 Czestochowa, Poland; marek.gala@pcz.pl

**Keywords:** steel section, heat treatment, complex heat transfer, thermo-electric analogy, thermal resistance

## Abstract

During the heat treatment of square or rectangular steel sections, a heated charge, arranged in regular packages, is placed inside a furnace. This type of charge forms a porous medium through which a complex heat flow occurs during heating. Several heat transfer mechanisms act simultaneously within this medium: conduction through the section walls, conduction and natural convection within the gas, thermal radiation between the section walls, and complex heat transfer (mainly contact conduction) at the joints between the adjacent sections. This article presents a qualitative analysis of heat transfer, aiming to determine the contribution of individual heat transfer mechanisms to the overall process. For this purpose, an analytical model of complex heat transfer within the package was employed, based on the thermo-electric analogy. The results from experimental studies were used to calculate the natural convection and heat transfer at the joints. It was assumed that the material of the sections was low-carbon steel, and the gas was air. Calculations were performed for the temperature range of 25 °C to 700 °C, considering three different geometrical configurations of the sections. It was shown that the effective thermal conductivity (ETC) of the package for the considered geometrical cases varies between 2.2 and 10.6 W/(m·K), which is an order of magnitude lower than the thermal conductivity of the individual sections. This parameter increased dynamically with the temperature. Moreover, the heat transfer intensity within the package of sections was nearly an order of magnitude lower than the heat conduction observed in a solid steel charge. Additionally, it was shown that the primary heat transfer mechanisms governing the heating process were thermal conduction (in the lower temperature range—up to approximately 350 °C) and thermal radiation (in the higher temperature range—above 350 °C). The gas convection inside the sections had a minimal impact on the heating process of the package. The primary parameters influencing the quality of the results were the joint resistance between the adjacent sections and the emissivity of the sections. The presented model can be used for the optimization of heat treatment processes for the considered charge.

## 1. Introduction

**Figure 1:**
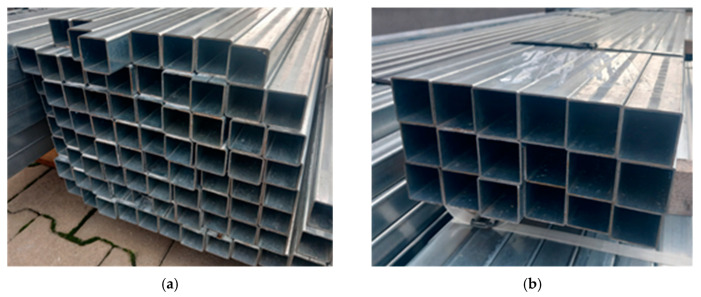
Packages of steel square sections prepared for heat treatment. (**a**) sections 60 mm; (**b**) sections 80 mm.

In response to the climate crisis, manifesting itself as global warming, various industrial sectors are actively developing technologies aimed at reducing energy consumption and greenhouse gas emissions [1,2,3,4,5,6]. This challenge also affects the metallurgical sector, particularly in the heat treatment of steel products [7,8,9,10,11]. Additionally, industrial heat treatment operations significantly impact production efficiency and the quality of finished products. For this reason, ongoing research addresses various aspects of the heat treatment of steel products [12,13,14,15,16,17]. These challenges make it essential to optimize such processes. It should be emphasized that this optimization should occur both at the process design stage and during its execution on the production line. Today, for efficient process design and control, specialized numerical models are employed, capable of predicting spatial and temporal temperature changes in the treated charge. Such models have been successfully used in the metallurgical industry for over four decades [18,19,20]. One of the crucial parameters for the accurate operation of these models is the thermal properties of the heated charge. When analyzing the heating process of monolithic steel elements, the primary thermal property is the thermal conductivity of steel. This task becomes especially challenging when dealing with a porous structure within the treated charge [21,22,23]. An example of such a charge is a package of square or rectangular steel sections (Figure 1). The inhomogeneous, two-phase structure (steel–gas) of these elements leads to complex heat flow dynamics, arising from the simultaneous occurrence of heat conduction, natural convection, and thermal radiation. For this reason, the thermal property essential for optimizing heat treatment is not the thermal conductivity of steel itself. Instead, the effective thermal conductivity (ETC or *k_ef_*), as the primary thermal property of section bundles, should be considered. This parameter is widely used in studies of porous media [24,25,26]. By employing effective thermal conductivity, it becomes possible to describe transient heat transfer within a porous charge and to determine its heating time [23,27,28], which is crucial for optimizing heat treatment processes.

The most reliable method for determining effective thermal conductivity is through experimental investigations. For cellular and porous materials, this is typically measured using the steady-state method with a guarded hot plate apparatus [29,30]. However, this approach requires specialized equipment, is time-consuming, and involves preparing specific test samples. A major limitation of these measurements is that the results are not universally applicable, as they are specific to individual material samples. Consequently, model calculations are often used as an effective alternative for determining the ETC of porous materials [25].

Various analytical models for estimating the effective thermal conductivity of two-phase media (solid–gas) are available in the literature. The most commonly used ETC models include the following: parallel and series models [31], Effective Medium Theory (EMT) [32], Maxwell–Eucken [32], Horai [33], Beck [34], Krischer [35], Woodside–Messmer [36], Assad [37], and Bruggeman [38]. However, as demonstrated in our calculations, these models are not suitable for determining the ETC of section bundles [39]. This limitation arises because these models do not account for the specific heat transfer mechanisms occurring during the heating of the charge in question, particularly contact conduction and thermal radiation.

This article presents a mathematical model for the effective thermal conductivity of a package of square steel sections. It is an empirical model because the relationships describing thermophysical parameters—such as the thermal conductivity of steel and gas, the Nusselt number, and joint resistance—were determined based on the approximation of experimental data. The results of these studies have been detailed in previous publications by authors [40,41,42,43]. This model is relatively simple yet provides a comprehensive description of the complex heat transfer phenomena occurring within the package of sections.

The greatest scientific contribution of the presented research lies in demonstrating that, thanks to the developed computational model, it is possible to quantitatively and qualitatively analyze the highly complex physical phenomenon of heat transfer using relatively simple mathematical relationships. The primary scientific objective of this article is to illustrate to what extent the heating process of a package of sections differs from the heating of solid steel elements, and to identify which heat transfer mechanisms primarily determine its intensity.

## 2. Materials and Methods

The presented model is based on the analysis of thermal resistances associated with individual heat transfer mechanisms occurring during the heating of the considered medium. This approach relies on the analogy between electrical and thermal conduction phenomena, stemming from the similarity in the mathematical expressions of Ohm’s and Fourier’s laws [44]. In many cases, this method provides an effective alternative for addressing complex heat transfer problems in heterogeneous systems, offering a simpler solution than more complex numerical methods [45,46,47,48,49,50,51,52].

**Figure 2:**
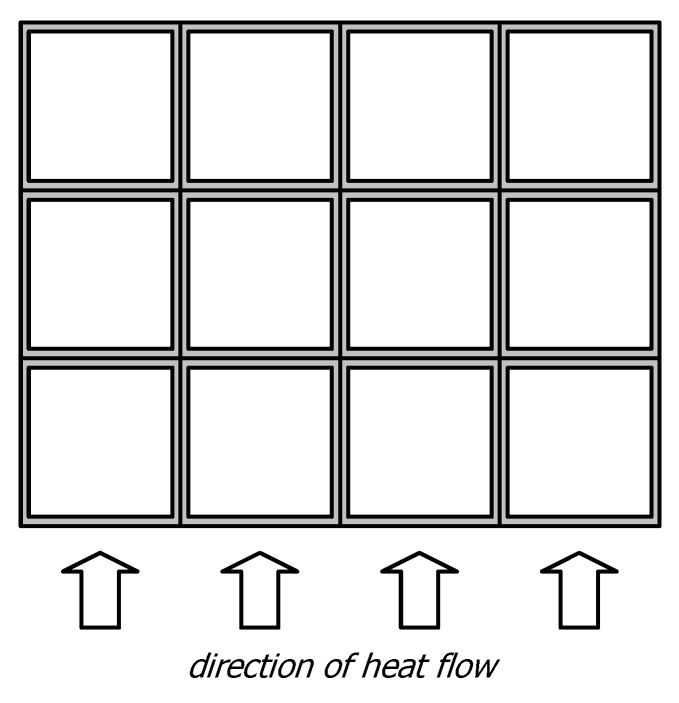
The physical model of the system under consideration in the form of a layered, packed bed of square sections with a steady, unidirectional heat flow.

The starting point for deriving the appropriate mathematical relationships that describe the phenomenon of complex heat flow was the physical model of the medium under consideration. As shown in Figure 2, this model consists of a packed, layered bed of square sections, within which unidirectional, steady heat flow occurs. Due to this assumption, thermal energy in this system is transferred alternately through the layers of sections and the joints between them.

The value of ETC for the considered medium was calculated using the definition of thermal resistance to conduction through the plane wall [53]:

(1) $$ETC=\frac{\delta_{c}}{R_{to}},$$

where *R_to_* and *δ_c_* are the total thermal resistance and characteristic dimension of the considered medium, respectively. Since the width of the joints was on the order of hundredths of a millimeter, it was assumed that the parameter *δ_c_* corresponded to the external dimension of the sections.

The total thermal resistance of the considered bed was a serial connection of section thermal resistance *R_st_* and joint thermal resistance *R_j_*:

(2) $$R_{to}=R_{st}+R_{j},$$

In order to determine the resistance *R_st_*, it was necessary to take into consideration the following phenomena of heat transfer: conduction in steel walls, conduction and free convection within gas (these two phenomena were analyzed jointly) and the thermal radiation between the inner surfaces of a section. Each of the mentioned mechanisms was assigned a corresponding thermal resistance, i.e., thermal resistance in steel, thermal resistance in gas *R_gs_* and radiation resistance *R_rd_*.

**Figure 3:**
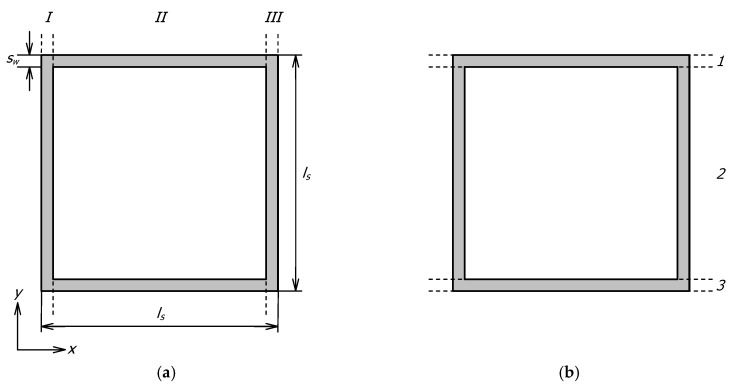
Division of a section into: (**a**) vertical zones I–III, (**b**) horizontal layers 1–3.

**Figure 4:**
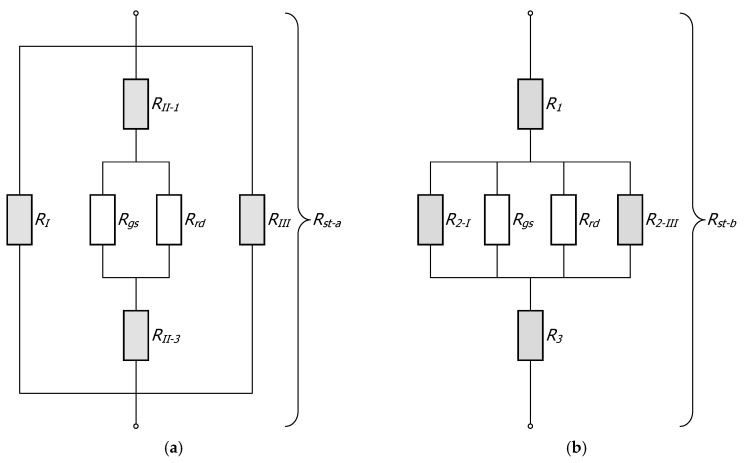
Thermal resistance networks of a square section: (**a**) network for resistance *R*_st-a_, (**b**) network for resistance *R*_st-b_.

Since the actual, multidimensional phenomenon of heat transfer was treated here as one-dimensional, obtaining a solution required two assumptions: (a) any plane wall normal to the direction of heat flow was isothermal, and (b) any plane parallel to the direction of heat flow was adiabatic [44]. This led to two separate resistance networks, resulting in two different total thermal resistances denoted as *R_st-a_* and *R_st-b_*. The resistance *R_st-a_* corresponded to the division of the section into three vertical zones I–III, parallel to the direction of heat flow, as shown in Figure 3a. The resistance *R_st-b_* corresponded to the division of the section into three horizontal layers 1–3 (Figure 3b). The thermal resistance networks corresponding to the different section divisions are presented in Figure 4.

Taking into account the adopted assumptions, the resistances *R_st-a_* and *R_st-b_* could be described using the following relationships:

(3) $$R_{st-a}={\left(\frac{1}{R_{I}}+\frac{1}{R_{II-1}+{\left(\frac{1}{R_{gs}}+\frac{1}{R_{rd}}\right)}^{-1}+R_{II-3}}+\frac{1}{R_{III}}\right)}^{-1},$$

(4) $$R_{st-b}=R_{1}+{\left(\frac{1}{R_{2-I}}+\frac{1}{R_{gs}}+\frac{1}{R_{rd}}+\frac{1}{R_{2-III}}\right)}^{-1}+R_{3}$$

The final value of the *R_st_* was calculated using the following formula [54]:

(5) $$R_{st}=\frac{R_{st-a}+2R_{st-b}}{3},$$

The resistances from Equations (3) and (4), which relate to conduction in section walls (they were marked gray in Figure 4), were described with the following relationships:

(6) $$R_{I}=R_{III}=\frac{l_{s}}{k_{s}\cdot f_{I}},$$

(7) $$R_{II-1}=R_{II-3}=\frac{s_{w}}{k_{s}\cdot f_{II}}$$

(8) $$R_{1}=R_{3}=\frac{s_{w}}{k_{s}}$$

(9) $$R_{2-I}=R_{2-III}=\frac{\left(l_{s}-2s_{w}\right)}{k_{s}\cdot f_{I}}$$

where *l_s_* is the external dimension of the section, *s_w_* is the wall thickness of the section, *k*_s_ is the thermal conductivity of steel, and *f_I_*, *f_II_* and *f_III_* are the relative surface areas of the respective zones:

(10) $$f_{I}=f_{III}=\frac{s_{w}}{l_{s}},$$

(11) $$f_{II}=\frac{l_{s}-2s_{w}}{l_{s}}$$

The resistance *R*_gs_ pertains to the heat transfer processes in the internal area of the section that is filled with gas. These processes include heat conduction and free convection. In the adopted solution, both phenomena were treated jointly as an intensified conduction, expressed quantitatively by the equivalent gas thermal conductivity *k_eq_* [44]:

(12) $$k_{eg}=Nu\cdot k_{g},$$

where *Nu* is the Nusselt number and *k_g_* is the thermal conductivity of gas.

The internal spaces of the sections were treated as horizontal enclosures. For this type of geometry, the Nusselt number was calculated using the criterial relationship with the Rayleigh number *Ra* [54]:

(13) $$Nu=C\cdot Ra^{m},$$

where the constants *C* and *m* depended on the Rayleigh number *Ra* (the values of *C* and *m* used are listed in Table 1).

Based on the research described in [42,43], for calculating the temperature change in the Nusselt number for square sections (60 mm and 80 mm), the following relationships were established:

(14) $$Nu_{60}=4.3\cdot 10^{-13}t^{5}-7.9\cdot 10^{-10}t^{4}+6.1\cdot 10^{-7}t^{3}-2.11\cdot 10^{-4}t^{2}+0.027\;t+2.6,$$

(15) $$Nu_{80}=6.1\cdot 10^{-13}t^{5}-1.3\cdot 10^{-9}t^{4}+1.05\cdot 10^{-6}t^{3}-3.96\cdot 10^{-4}t^{2}+0.056\;t+4.2$$

As demonstrated in the referenced studies, for sections smaller than 60 mm, the Nusselt number did not exceed the value of 1.2. Therefore, for such sections, the phenomenon of free convection was neglected. Ultimately, the resistance *R_gs_* was described by the formula:

(16) $$R_{gs}=\frac{\left(l_{s}-2s_{w}\right)}{Nu\cdot k_{g}\cdot f_{II}},$$

Radiation resistance *R_rd_* was solved through analysis of the radiosity balance of flat surfaces in the square cavity that closed a space [55]. The methodology for the determination of resistance *R_rd_* for such geometry was described in [56]. When the temperature difference Δ*T* in the system was much smaller than its mean absolute temperature *T*, radiation resistance could be described by:

(17) $$R_{rd}=\frac{1}{\;\varepsilon }\cdot \frac{1}{4\sigma_{c}\;T^{3}},$$

where *σ*_c_ is the Stefan–Boltzmann constant and *ε* is the emissivity of the section surface.

According to Equation (2), the intensity of heat flow in the section package also depended on the joint thermal resistance *R_j_*. Due to the shape inaccuracies of the sections, contact between the adjacent layers did not occur across the entire surface of the package, but only on small discrete contour areas whose geometry was entirely random. For this reason, the resistance *R_j_* could not be described by commonly used analytical relationships [57,58,59,60]. Furthermore, experimental investigations of this parameter are also challenging due to the hollow interior of the sections, which complicates the measurement of temperature distribution along those elements. To overcome this problem, it was assumed that the joint resistance in this case was similar to the joint resistance in a bed of rectangular steel bars. As established through experimental measurements using a guarded hot plate apparatus, the joint resistance for this type of medium decreased with increasing temperature [40]. Moreover, the variability of many physical factors characterizing the discussed joints caused the resistance *R_j_* at a given temperature to take on values within a certain range. The minimum and maximum values of the resistance *R_j_* in the function of temperature could be described by the following formulas:

(18) $$R_{j-\min}=\left(1.25\cdot 10^{-5}\;t^{2}-0.0288\;t+32.91\right)\cdot 10^{-4},$$

(19) $$R_{j-\max}=\left(2.31\cdot 10^{-5}\;t^{2}-0.0534\;t+58.16\right)\cdot 10^{-4}$$

As indicated by Equations (18) and (19), in the temperature range of 25 to 700 °C, the value of *R_j-max_* constituted approximately 180% to 143% of the value of *R_j-min_*.

It should be noted that the proposed model also allowed for the analysis of heat transfer in a package of rectangular profiles. In this case, instead of a single profile dimension *l_s_*, two dimensions must be provided (width *l_x_* and height *l_y_*). For rectangular geometry, the equations involving the *l_s_* parameter, as well as Equation (17), which described radiation resistance, were slightly modified. For rectangular geometry, this equation takes the form:

(20) $$R_{rd}=\frac{\left(\left(2l_{x}-4s_{w}\right)\cdot \frac{1}{\varepsilon }\right)+l_{y}-l_{x}}{\;\left(l_{x}+l_{y}-4s_{w}\right)}\cdot \frac{1}{4\sigma_{c}\;T^{3}}.$$

The first term on the right side of the above equation results from the algebra of view factors [56]. When the profile is square (*l_x_* = *l_y_* = *l_s_*), the above equation simplifies to form (17).

In summary, discussing the proposed mathematical model, it is important to emphasize that it describes the entire spectrum of thermal phenomena occurring during the heating of a package of sections, while also accounting for the variation in their intensity with increasing or decreasing temperature. It should also be noted that the originality of this model lies in the use of empirical equations, the forms of which the authors developed based on their own experimental research (Equations (14), (15), (18) and (19)) or by approximating the literature data (Equations (20) and (21)). As a result, it served as a highly versatile computational tool, enabling both quantitative and qualitative analyses of the heat transfer process within the package of steel sections. Furthermore, due to the simplicity of the mathematical relationships employed, calculations could be performed using standard spreadsheet software. To perform the calculations, the authors implemented this model in Scilab (version 6.0.1), a free and open-source software for engineers and scientists available under the GPL License. More information about this software can be found on the following website: https://www.scilab.org (accessed on 25 October 2024).

## 3. Results and Discussion

The analyses presented below pertain to heat transfer phenomena in a package of square profiles during annealing. This type of heat treatment aims to alter the structure of steel and its mechanical properties. In the case of carbon steel, annealing serves to reduce hardness, improve ductility, eliminate internal stresses, and homogenize the material structure. The temperature at which these processes occur depends on the type of annealing and the carbon content in the steel [61]. For example, softening annealing is conducted at temperatures between 650 and 750 °C (below the critical temperature of steel) and aims to reduce hardness, facilitating further mechanical processing. Stress-relief annealing occurs at lower temperatures, typically between 550 and 650 °C, and aims to reduce internal stresses that may have developed due to previous mechanical or thermal processing. The steel is slowly cooled after being heated to this temperature. It is important to note that the exact annealing temperature depends on the carbon content of the steel. Steel with a higher carbon content (e.g., high-carbon steel) will have higher austenitizing temperatures compared to low-carbon steel. All the calculations were conducted for a temperature range of 25 to 700 °C. As indicated by the information provided above, this is the typical temperature range encountered during the annealing of carbon steel products.

In the calculations, the following parameters were used as the input data: section size *l_s_*, section wall thickness *s_w_*, section surface emissivity *ε* (assumed *ε* = 0.7), Nusselt number *Nu*, joint thermal resistance (minimum *R_j-min_* and maximum *R_j-max_*), and the thermal conductivities of steel *k_s_* and gas *k_g_*. It was considered that the thermophysical parameters *Nu*, *R_j_*, *k_s_*, and *k_g_* vary with temperature. Changes in the thermal conductivity of steel over the temperature range of 25 to 700 °C were described by the following equation [62]:

(21) $$\begin{array}{c} k_{s}=112.51x_{c}^{2}-100.85\;x_{c}+68.89-\left(0.42x_{c}^{2}+0.318\;x_{c}+0.065\right)\;t\\ +\left(334.05x_{c}^{2}-247.27\;x_{c}+18.59\right)\cdot 10^{-6}\;t^{2} \end{array},$$

where *x_c_* means the percentage of carbon in the steel (it was assumed *x_c_* = 0.2%).

In performing the calculations, it was assumed that the gas filling the interior of the sections was air. The equation describing the changes in the thermal conductivity of air over the temperature range of 25 to 700 °C was derived by approximating tabulated data [63]:

(22) $$k_{g-air}=-2.88\cdot 10^{-8}t^{2}+8.05\cdot 10^{-5}t+0.024,$$

Model calculations illustrating various aspects of the heat transfer phenomenon within the package of sections were performed for nine geometric cases. Three section sizes *l_s_* were considered: 40 mm, 60 mm, and 80 mm. For each section, three wall thicknesses *s_w_* were taken into account: 1 mm, 2 mm, and 3 mm for the 40 mm section, and 2 mm, 3 mm, and 4 mm for the 60 mm and 80 mm sections. Additionally, for each case, calculations were performed for both the minimum and maximum values of the joint resistance *R_j_*. In the first case, the maximum values of the effective thermal conductivity (ETC) were obtained (ETC^max^), while in the second case, the minimum values were obtained (ETC^min^).

**Figure 5:**
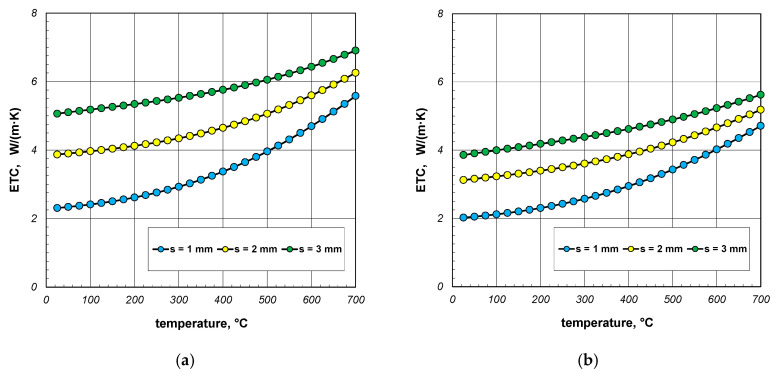
ETC of the 40 mm section package for the (**a**) minimum and (**b**) maximum joint resistance.

**Figure 6:**
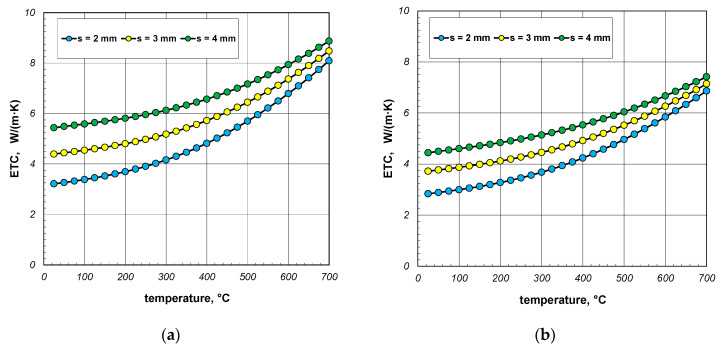
ETC of the 60 mm section package for the (**a**) minimum and (**b**) maximum joint resistance.

**Figure 7:**
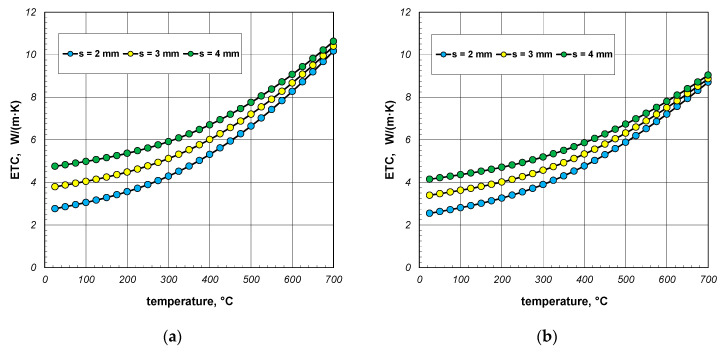
ETC of the 80 mm section package for the (**a**) minimum and (**b**) maximum joint resistance.

Firstly, the results of the calculations of effective thermal conductivity (ETC) for the case of complete heat exchange, when all the possible mechanisms of this phenomenon (conduction, free convection, and radiation) occurred, are shown. These results, in the form of graphs showing changes in the considered parameter as a function of temperature, were divided according to the various section sizes and are presented in Figure 5, Figure 6 and Figure 7. The graphs labeled (a) show the maximum values of ETC, while the graphs labeled (b) show the minimum values. Each graph presents three series of results referring to different wall thicknesses of the section. As evident from the provided graphs, the analyzed parameter increased as a function of the temperature in every case. This trend was attributed to the phenomenon of thermal radiation, the intensity of which was proportional to the third power of the absolute temperature. The increase in ETC also occurred with larger section dimensions and greater wall thickness. The first effect was caused by the interplay of many factors, with thermal radiation having the most significant influence. The second effect arose from the increased contribution of heat conduction through the walls of the sections. Given that steel is a good conductor of heat, this mode of heat transfer was the most effective mechanism for transferring energy within the examined charge.

The results of the calculations presented in the graphical form primarily demonstrated the qualitative nature of the changes in the ETC parameter. However, to better convey the computational data in a quantitative sense, they were also presented in a tabular form (Table 2), where for each computational case, the minimum, average, and maximum values of the ETC were compiled.

As indicated by the results presented above, increasing the external dimension of the profiles *l_s_* and the thickness of their walls *s_w_* led to a rise in the value of the effective thermal conductivity (ETC). An increase in the parameter *l_s_* resulted in fewer joints per unit length of the bundle, which corresponded to the unit of the ETC. These joints represented the areas of highest thermal resistance. Therefore, if the increase in *l_s_* reduced the number of joints, the overall thermal resistance of the medium decreased, thereby increasing the value of the ETC. Conversely, increasing the wall thickness enhanced the area for heat conduction in the steel, which increased the parameters *f_I_* and *f_III_*, consequently reducing the thermal resistances *R_I_* and *R_III_* described by Equation (6).

A significant issue in analyzing heat flow within the package of sections was to decipher to what extent the value of the ETC differed from the thermal conductivity of the steel from which the sections were made. The difference between these two parameters straightforwardly indicated how quantitatively the heat transfer process in the package deviated from the simple heat conduction in solid steel. This discrepancy was illustrated using a parameter called the reduced effective thermal conductivity *R_ETC_*, which is defined as the following product:

(23) $$R_{ETC}=\frac{ETC}{k_{s}},$$

**Figure 8:**
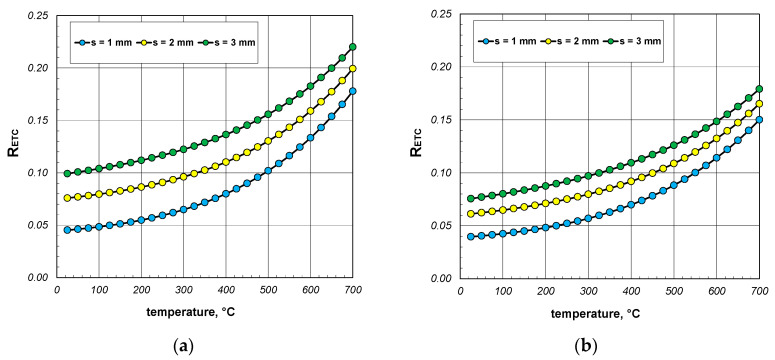
*R_ETC_* of the 40 mm section package for the (**a**) minimum and (**b**) maximum joint resistance.

**Figure 9:**
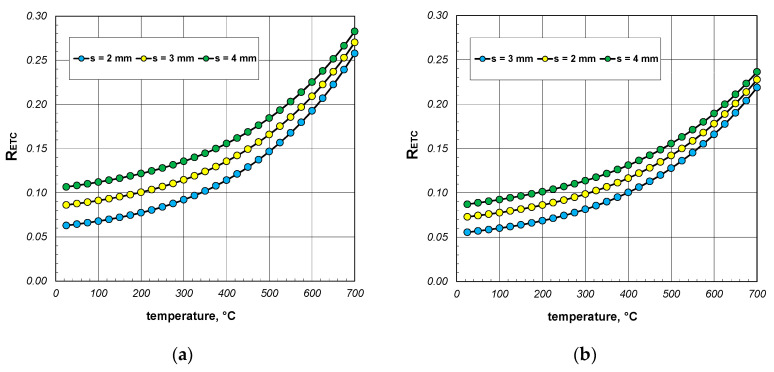
*R_ETC_* of the 60 mm section package for the (**a**) minimum and (**b**) maximum joint resistance.

**Figure 10:**
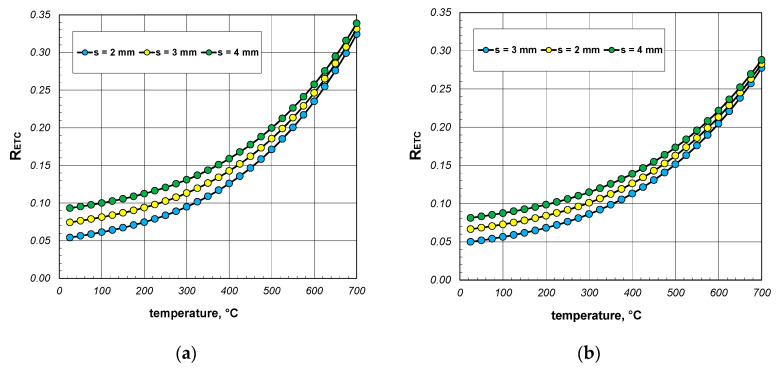
*R_ETC_* of the 80 mm section package for the (**a**) minimum and (**b**) maximum joint resistance.

The results of the calculations for the *R_ETC_* parameter, presented in the form of graphs for each case of the analyzed charge, are shown in Figure 8, Figure 9 and Figure 10. As seen, the *R_ETC_* parameter increased significantly as a function of the temperature. The same effect was observed with the increasing section dimensions and wall thickness. Generally, for all the cases of the charge, the value of *R_ETC_* ranged from 0.04 to 0.34, with an average value of approximately 0.15. This value indicates that the intensity of heat flow in the package of sections was nearly an order of magnitude lower than that of the conduction in steel (in the walls of the sections). This highlights how distinct the physical process of heating a package of sections, which is a porous granular material, is compared to the heating of solid steel charges. As with the ETC, for each computational case, Table 3 presents the minimum, average, and maximum values of *R_ETC_*.

The results presented in the following sections of the article pertain to the qualitative analysis of the heat transfer process within the package of sections. First, the aim was to determine the impact of the heat transfer phenomena occurring in the gas filling the interiors of the sections. As previously mentioned, these phenomena include conduction and free convection. To analyze this problem, a parameter denoted as *MG_ETC_* was utilized, which is defined as the following quotient:

(24) $$MG_{ETC}=\frac{ETC-ETC_{WG}}{ETC_{WG}},$$

where ETC_WG_ denotes the effective thermal conductivity of the package calculated by excluding the heat transfer phenomena occurring in the gas region. The value of ETC_WG_ was obtained by omitting the resistance *R_gs_* in the computational model.

**Figure 11:**
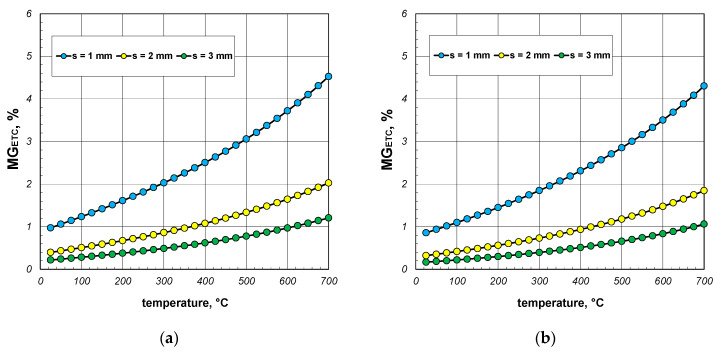
MG_ETC_ parameter of the 40 mm section package for the (**a**) minimum and (**b**) maximum joint resistance.

**Figure 12:**
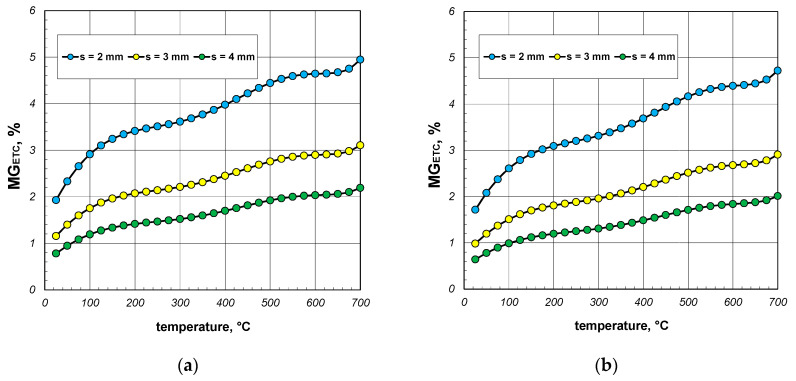
MG_ETC_ parameter of the 60 mm section package for the (**a**) minimum and (**b**) maximum gap resistance.

**Figure 13:**
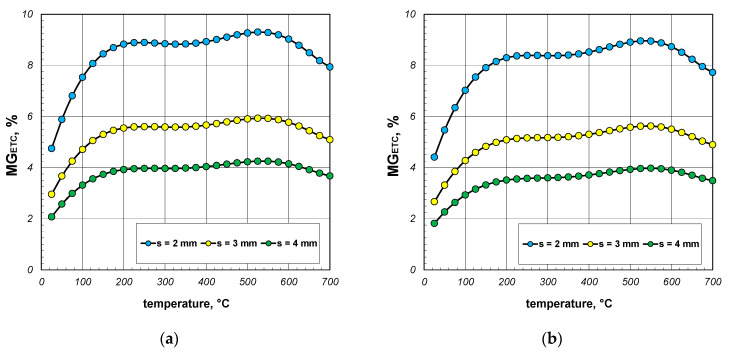
MG_ETC_ parameter of the 80 mm section package obtained for the (**a**) minimum and (**b**) maximum gap resistance.

The results of the *MG_ETC_* parameter calculations are presented in Figure 11, Figure 12 and Figure 13. When discussing these data, it is important to note that the free convection of air in enclosures is highly dependent on their geometry (dimensions and shape). In this analyzed case, these enclosures were horizontal channels with a square cross-section. As determined by our own research, convection was practically non-existent in the 40 mm sections [41,42]. Therefore, it was assumed that for this package of sections, the Nusselt number was constant and took a value of 1.2. For the packages of the 60 mm and 80 mm sections, the Nusselt number was calculated using Equations (14) and (15). As a result, the changes in the *MG_ETC_* parameter obtained for the various packages were highly varied. In the case of the 40 mm section package (Figure 11), where free convection was negligible, the analyzed parameter increased as a function of the temperature. This trend in the results was attributed to the changes in the thermal conductivity of both the steel and the air. Within the analyzed temperature range, the coefficient *k_s_* decreased from 50.1 to 29.4 W/(m·K), while *k_g_* increased from 0.026 to 0.061 W/(m·K). The *MG_ETC_* parameter decreased significantly with the increasing wall thickness of the section. In summary, it can be stated that for the 40 mm section package, the heat transfer phenomena in the gas accounted for a maximum of 4% of the energy transfer, with an average value of approximately 1%.

A different trend in the *MG_ETC_* parameter was observed for the packages of the 60 mm and 80 mm sections. In this case, the free convection of air was significantly developed, resulting in higher values of the Nusselt number. The maximum values of the Nusselt numbers were 4.9 for the 60 mm sections and 7.1 for the 80 mm sections, occurring at approximately 80 °C. Further increases in the temperature led to a linear decrease in the Nusselt number, which reached around 2 at a temperature of 700 °C for both of the sections.

In the case of the 60 mm section package (Figure 12), the *MG_ETC_* parameter increased across the entire temperature range, with the lines illustrating changes in this parameter showing slight fluctuations. For this charge, the convection and conduction in the gas transfer reached a maximum of approximately 5% of the energy, with an average value of about 2.5%. For the 80 mm section package (Figure 13), the *MG_ETC_* parameter rose significantly up to about 200 °C, after which its value stabilized until approximately 550 °C. With further increases in the temperature, a decline in this parameter was observed. In this case, the maximum *MG_ETC_* value reached around 9%, with the average value stabilizing at about 5%. Table 4 summarizes the minimum, average, and maximum *MG_ETC_* values obtained for all the considered cases. To conclude this part of the research, it should be noted that the thermal phenomena occurring in this gas region had a negligible impact on the overall heat transfer during the heating and cooling of the discussed charge. Ignoring these phenomena greatly simplified the mathematical description of the heat exchange process without adversely affecting the accuracy of the obtained results.

Another step of the quantitative analysis of heat exchange in the section package was to examine the impact of thermal radiation. To analyze this problem, a parameter denoted as *MR_ETC_* was defined as follows:

(25) $$MR_{ETC}=\frac{ETC-ETC_{WR}}{ETC_{WR}},$$

where ETC_WR_ denotes the effective thermal conductivity of the package calculated by neglecting the thermal radiation between the internal surfaces of the section. The value of ETC_WR_ was obtained by excluding the radiation resistance *R_rd_* from the computational model.

**Figure 14:**
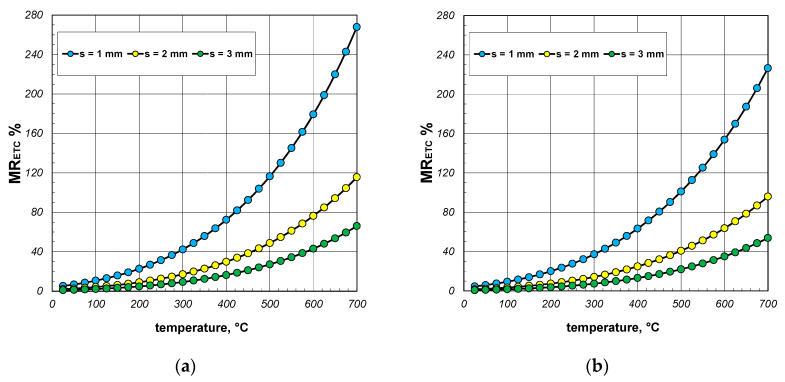
MR_ETC_ parameter of the 40 mm section package for the (**a**) minimum and (**b**) maximum gap resistance.

**Figure 15:**
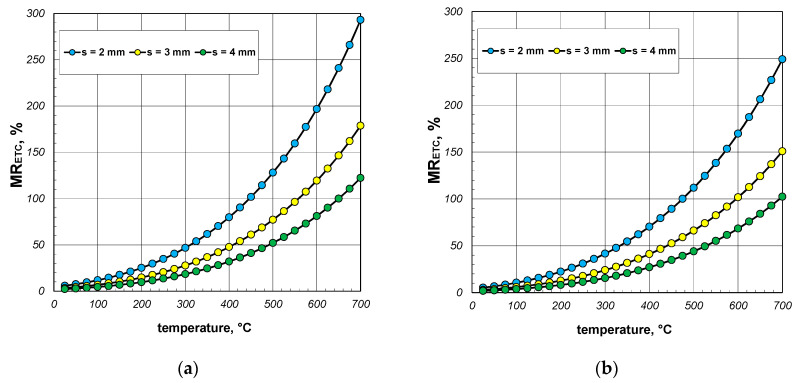
MR_ETC_ parameter of the 60 mm section package for the (**a**) minimum and (**b**) maximum gap resistance.

**Figure 16:**
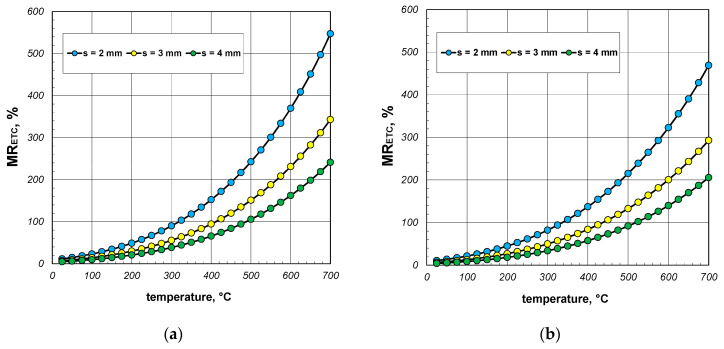
MR_ETC_ parameter of the 80 mm section package for the (**a**) minimum and (**b**) maximum gap resistance.

The results of the calculations for the parameter *MR_ETC_* are presented in Figure 14, Figure 15 and Figure 16. As seen in all the cases, this parameter increased significantly with temperature. This trend arose from the fact that the intensity of the thermal radiation between the internal surfaces of the section was proportional to the third power of their mean absolute temperature. In contrast to the parameter *MG_ETC_*, which remained at a few percent, the values of *MR_ETC_* significantly exceeded 100%. Up to a temperature of 400 °C, the heating of the package was primarily driven by heat conduction through the walls of the sections. However, beyond this temperature, thermal radiation became the key phenomenon responsible for the transfer of thermal energy. Thus, when analyzing the heat transfer process in the examined load, neglecting thermal radiation was not an option. The minimum, average, and maximum values of *MR_ETC_* obtained for all the analyzed cases are summarized in Table 5.

**Figure 17:**
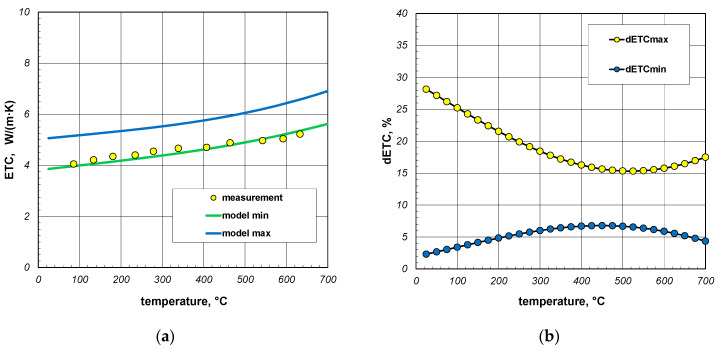
Comparison of measured and model values of ETC for 40 mm sections package (**a**) and changes in dETC parameter (**b**); results obtained for emissivity 0.7.

**Figure 18:**
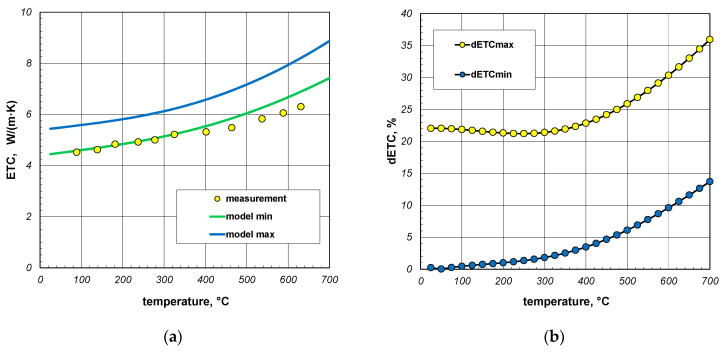
Comparison of measured and model values of ETC for 60 mm sections package (**a**) and changes in dETC parameter (**b**); results obtained for emissivity 0.7.

The final element of the analysis presented in this article was to assess the quality of the results from the proposed model. For this purpose, the results of the experimental investigations on the effective thermal conductivity (ETC) of the square section packages were utilized [64]. The samples used in the experiments were constructed from sections made of S235JRH steel grade, which had a maximum carbon content of 0.2% [65]. The measurement results obtained for the 40 mm and 60 mm sections with a wall thickness of 3 mm are shown in Figure 17a and Figure 18a, respectively. These figures also include the model calculation results obtained for the minimum and maximum values of resistance *R_j_*. As can be seen, in both cases, the measured results were close to the minimum model values. To perform a precise quantitative comparison of the presented results, the measurement data were approximated with regression functions in the form of second-degree polynomials. For the individual sections, these functions are as follows:

(26) $$ETC_{40}=6.3\cdot 10^{-7}\cdot t^{2}+0.0024t+3.89,$$

(27) $$ETC_{60}=2.3\cdot 10^{-6}\cdot t^{2}+0.0014t+4.42.$$

The determination coefficients *R_2_* for these functions were 0.986 and 0.984, respectively. Values close to unity indicate that the adopted equations are well-fitted to the measurement results.

For further analysis, the parameter dETC was defined using the following equations:

(28) $$dETC_{\max}=\frac{\left|ETC_{me}-ETC_{\max}\right|}{ETC_{me}}\cdot 100\%$$

(29) $$dETC_{\min}=\frac{\left|ETC_{me}-ETC_{\min}\right|}{ETC_{me}}\cdot 100\%$$

where ETC_me_—measured values, ETC_max_—maximum modeled values, ETC_min_—minimum modeled values.

The results of the calculations for the parameter dETC obtained for the analyzed packages are presented in Figure 17b and Figure 18b. For the 40 mm profile package, the average value of the parameter dETCmin was 5.3%, while the maximum value was 6.8%. This indicates a relatively high consistency between the model results and the measurement data. For the parameter dETCmax, these values were 19.1% and 28.2%, respectively. For the 60 mm profile package, the average and maximum values of dETCmin were 4.4% and 13.7%, respectively. For dETCmax, the values were 24.8% and 35.9%. The presented results demonstrate that when modeling the ETC of the profile package, it was necessary to use Equation (19), which describes the maximum value of this parameter, when addressing the thermal resistance of joints. The ETC values calculated using Equation (18), which described the minimum value of *R_j_*, were overestimated for both profile dimensions.

**Figure 19:**
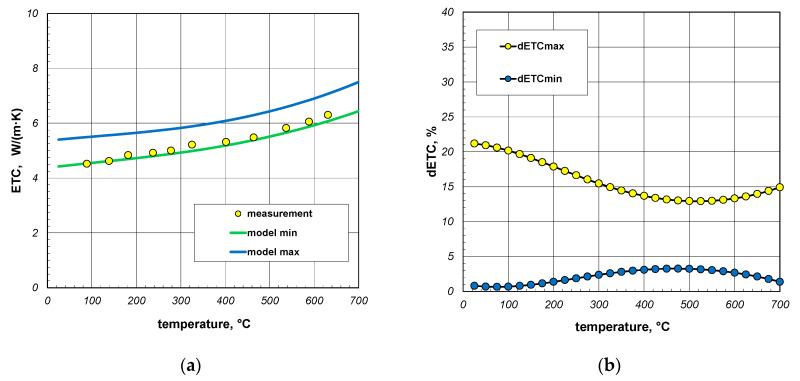
Comparison of measured and model values of ETC for 60 mm sections package (**a**) and changes in dETC parameter (**b**); results obtained for emissivity 0.5.

As shown in Figure 18a, the measurement results and minimum model values were practically coincident up to a temperature of about 350 °C. However, with further increases in temperature, a growing difference was observed, with the model values exceeding the measurement data. Consequently, additional calculations were performed with a reduced emissivity of 0.5. This emissivity was characteristic of cold-rolled steel products with a clean surface [66,67]. The results of these calculations are presented in Figure 19a. In this situation, the model values were slightly lower than the measurement data. Considering that the experimental data were characterized by a measurement uncertainty of 5% [64], it can be concluded that, in this case, a nearly perfect fit had been achieved for the minimum model values. This applies to both the values of ETC and the nature of their changes as a function of temperature. As shown in Figure 19b, the maximum value of the parameter dETCmin for this case was 3.8%, with an average value of 2.1%. This was below the measurement uncertainty of the experimental results.

The results presented demonstrate that using the proposed computational model, along with the proper adjustment of the thermophysical parameters, it is possible to accurately replicate the values of the effective thermal conductivity (ETC) of the profile package. Given that the analyzed problem involved a very complex physical phenomenon, the behavior of which depended on a variety of factors, even an adjustment accuracy of several percentage points, as achieved for ETCmax, should be considered acceptable.

## 4. Conclusions

Based on the calculations performed, the following conclusions can be drawn:

- The effective thermal conductivity (ETC) of the package of square steel sections for the considered geometrical cases varied between 2.2 and 10.6 W/(m·K);
- The ETC value of the package was an order of magnitude lower than the thermal conductivity of the individual sections;
- The ETC of the analyzed charge increased dynamically with temperature;
- The ETC increased as a function of the section size and wall thickness;
- Below 350 °C, the primary mechanism governing the heating of the package was heat conduction in the section walls;
- Above 350 °C, the mechanism that determined the heating of the package was thermal radiation;
- The gas convection inside the sections had minimal impact on the heating process of the package;
- The key parameters influencing the quality of the results were the joint resistance between adjacent sections and the emissivity of the sections.

In summary, the presented research emphasizes that the proposed model is universal and allows for the analysis of various geometric configurations related to changes in the dimensions of the profiles, as well as the packing or arrangement of a bundle. However, for a package with different geometry, the form of the individual equations must be modified to account for new geometric parameters. The article only analyzed the impact of wall thickness and the external dimensions of the profiles on heat transfer in a maximally packed bundle, as this type of charge was most commonly subjected to heat treatment. A loosely packed bundle (with higher internal porosity due to the presence of gaps between the profiles) will exhibit a lower value of ETC and, consequently, will heat up more slowly. At the same time, it will occupy more space in the furnace chamber, which should be utilized as efficiently as possible to maximize production process efficiency. For this reason, it is rational to heat only maximally packed bundles.

This model can be applied to a bundle of profiles made from any material. When changing the material, it is only necessary to adjust the equations describing its thermal conductivity (Equation (20)) and the equations describing the resistance *R_j_* (Equations (18) and (19)). However, the validation of the model for a different material requires additional experimental investigations, as the forms of Equations (18) and (19) can only be determined through measurements. This model also allows for the inclusion of the type of atmosphere in which the heat treatment is carried out. The effect of the gas type on heat transfer within the bundle is accounted for by the heat transfer coefficient *k_g_*, which appears in Equation (16). This equation describes the thermal resistance in the gas region. In the simplest case, one value can be provided, e.g., 0.024 W/(m·K)—the value for air at room temperature. In a more precise solution, a dependence describing the changes in this parameter as a function of temperature should be provided. This was also the approach taken during the calculations, where the change in air thermal conductivity as a function of temperature was described using a second-degree polynomial—Equation (21). Changing the type of gas in the model calculations requires only modifying one equation. The form of this equation, describing the temperature dependence of the thermal conductivity of a specific gas, can be determined by approximating the literature data. For example, if the heat treatment is conducted in a hydrogen atmosphere, which is often encountered in industrial practice [8,27], the coefficient *k_g_* should be described by the following equation:

(30) $$k_{g}=0.0004\;t+0.184.$$

In special cases, the heat treatment of steel products is conducted in vacuum furnaces [68,69]. The use of a vacuum offers several advantages, including the elimination of surface oxidation of the heated elements [70]. In the analysis of heat treatment in a vacuum furnace, the resistance *R_g_* described by Equation (16) should be omitted from the model. The impact of the gas type, or the absence of gas, on the heat transfer process within the bundle is an interesting problem and will undoubtedly be the subject of analysis in future publications.

As mentioned in the introduction, computer programs utilizing numerical algorithms are currently employed to control heat treatment processes. The presented model can be implemented as one of the procedures within such a program. The ability to accurately determine the thermophysical properties of the heated package provided by this model is crucial for optimizing the industrial heat treatment of steel sections.

## Figures and Tables

**Table 1:** Values of the constants *C* and *m* in relation to the Rayleigh number *Ra* [27].

Rayleigh Number *Ra*	*C*	*m*
10^4^ < *Ra* < 4 × 10^5^	0.195	0.25
4 × 10^5^ < *Ra* < 10^7^	0.068	0.33

**Table 2:** The minimum, average, and maximum values of ETC.

Section Size	Wall Thickness	ETC, W/(m·K)					
		R_j-min_			R_j-max_		
		Min	Mean	Max	Min	Mean	Max
40 mm	1 mm	2.31	3.47	5.58	2.02	3.01	4.71
	2 mm	3.87	4.72	6.26	3.12	3.91	5.18
	3 mm	5.06	5.71	6.91	3.85	4.61	5.62
60 mm	2 mm	3.21	4.96	8.09	2.83	4.32	6.86
	3 mm	4.39	5.84	8.48	3.72	4.99	7.14
	4 mm	5.44	6.67	8.87	4.44	5.58	7.42
80 mm	2 mm	2.77	5.48	10.18	2.55	4.87	8.71
	3 mm	3.80	6.19	10.40	3.39	5.44	8.87
	4 mm	4.76	6.87	10.63	4.15	5.97	9.04

**Table 3:** The minimum, average, and maximum values of *R_ETC_* parameter.

Section Dimension	Wall Thickness	R_ETC_					
		R_gp-min_			R_gp-max_		
		Min	Mean	Max	Min	Mean	Max
40 mm	1 mm	0.045	0.087	0.178	0.039	0.075	0.150
	2 mm	0.076	0.116	0.199	0.061	0.096	0.165
	3 mm	0.099	0.141	0.220	0.075	0.112	0.179
60 mm	2 mm	0.063	0.124	0.257	0.056	0.108	0.219
	3 mm	0.086	0.144	0.271	0.073	0.123	0.227
	4 mm	0.106	0.163	0.283	0.087	0.137	0.236
80 mm	2 mm	0.054	0.139	0.324	0.049	0.124	0.278
	3 mm	0.074	0.156	0.331	0.067	0.136	0.283
	4 mm	0.093	0.171	0.339	0.081	0.148	0.288

**Table 4:** The minimum, average, and maximum values of *MG_ETC_* parameter.

Section Dimension	Wall Thickness	MG_ETC_, %					
		R_gp-min_			R_gp-max_		
		Min	Mean	Max	Min	Mean	Max
40 mm	1 mm	0.97	2.47	4.53	0.86	2.28	4.30
	2 mm	0.39	1.07	2.03	0.32	0.93	1.85
	3 mm	0.22	0.62	1.21	0.16	0.51	1.06
60 mm	2 mm	1.93	3.82	4.94	1.71	3.53	4.72
	3 mm	1.16	2.35	3.11	0.93	2.11	2.90
	4 mm	0.78	1.63	2.19	0.64	1.42	2.01
80 mm	2 mm	4.75	8.45	9.30	4.41	8.05	8.96
	3 mm	2.96	5.36	5.93	2.66	4.99	5.62
	4 mm	2.07	3.81	4.25	1.82	3.49	3.97

**Table 5:** The minimum, average, and maximum values of *MR_ETC_* parameter.

Section Dimension	Wall Thickness	MR_ETC_, %					
		R_gp-min_			R_gp-max_		
		Min	Mean	Max	Min	Mean	Max
40 mm	1 mm	5.3	79.7	261.9	4.6	74.9	226.5
	2 mm	2.1	33.5	115.5	1.6	30.3	95.9
	3 mm	1.1	20.3	569.6	0.8	16.5	53.8
60 mm	2 mm	5.9	95.1	293.1	5.2	82.3	249.1
	3 mm	3.4	57.3	178.8	2.9	48.9	150.8
	4 mm	2.2	38.8	122.3	1.8	32.6	102.4
80 mm	2 mm	11.6	179.5	547.3	10.7	158.2	469.0
	3 mm	7.1	111.8	342.9	6.3	97.6	292.9
	4 mm	4.8	78.2	241.3	4.2	67.7	205.4

## Data Availability

The original contributions presented in this study are included in the article. Further inquiries can be directed to the corresponding author.
